# COVID-19 Pandemic Outbreak and its Psychological Impact on Patients with Rare Lysosomal Diseases

**DOI:** 10.3390/jcm9092716

**Published:** 2020-08-22

**Authors:** Agata Fiumara, Giuseppina Lanzafame, Alessia Arena, Annamaria Sapuppo, Federica Raudino, Andrea Praticò, Piero Pavone, Rita Barone

**Affiliations:** 1 Regional Referral Centre for Inborn Errors Metabolism, Pediatric Clinic, Department of Clinical and Experimental Medicine, University of Catania, 95123 Catania, Italy; giusylanz@virgilio.it (G.L.); alessia.arena@gmail.com (A.A.); annamaria.pan@hotmail.it (A.S.); federica.raudino@tiscali.it (F.R.); andrea.pratico@unict.it (A.P.); ppavone@unict.it (P.P.); 2 Child Neuropsychiatry, Department of Clinical and Experimental Medicine, University of Catania, 95123 Catania, Italy; rbarone@unict.it

**Keywords:** COVID-19 pandemic, ERT, lysosomal storage disease, psychological impact

## Abstract

Background: Lysosomal storage disorders (LSDs) are rare, chronic, progressive multisystem diseases implying severe medical issues and psychological burden. Some of these disorders are susceptible to a treatment, which is administered weekly or every other week, in a hospital. During the COVID-19 (Corona Virus Disease 2019) pandemic lockdown, patients with LSDs on enzyme replacement therapy (ERT) missed their scheduled access to the Day Hospital to get their treatment. Methods: Based on the feeling that our patients were experiencing profound distress, we designed a structured telephone interview with the aim to evaluate how, and to which extent, the pandemic outbreak was changing their behavior and feelings about their chronic disease, the impact on therapies, and future expectations. The same interview was administered to an age-matched control group. Results: All interviewed people experienced an increase of anxiety, worries, and uncertainty fostered by incessant media updates. Moreover, a striking similarity emerged between the groups regarding forced home reclusion and the profound feeling to be excluded by normal life, well-known to those affected by a chronic rare disease. Conclusions: Although no statistically significant difference was found compared to controls, we felt that the reactions were qualitatively different, underlining the fragility and isolation of such patients.

## 1. Introduction

Since March 2020, the dramatic outbreak of Corona Virus Disease 2019 (COVID-19) in Italy has changed our lifestyle as individuals, physicians, and patients. Despite the evidence of minor involvement of children [1], pediatric units also had to deal with healthcare crises.

As a Referral Centre for Inborn Errors of Metabolism (IEM), we had to face an unexpected restriction concerning daily normal activity with lowering of programmed admissions for diagnosis and follow-up visits. Our concern was especially directed to those patients with lysosomal storage disorders (LSDs), which are rare, chronic, progressive, multisystem diseases associated with serious medical issues, physical disability, and psychological burden [2]. In the last decade, some of the LSDs became treatable by pharmacological therapy, such as intravenous (iv) enzyme replacement therapy (ERT) and oral substrate reduction therapy (SRT), or chaperones. During the COVID-19 alert, patients with LSDs, under regular treatment with ERT, failed their usual compliant behavior, missing scheduled infusions. Based on the feeling that our patients were experiencing profound distress, we designed a structured interview [3,4] with the aim to evaluate how, and to which extent, the COVID-19 pandemic was changing our patients’ behavior and feelings about their chronic disease, the impact on therapies, and their future expectations. We emphasize the importance to investigate attitudes and behavior with respect to health treatment, especially among people with rare diseases, such as patients with LSD. They represent a group with increased vulnerabilities to COVID-19; thus, we felt the need to attempt any possible solution that would let them maintain treatment protocols and minimize disease progression. 

## 2. Methods

### 2.1. Study Design and Participants

At our Regional Referral Centre for Metabolic diseases, Pediatric Clinic, Department of Clinical and Experimental Medicine, patients with different types of IEM are followed. At the time of the study, 33 of them were affected by a treatable LSD and thus were regularly admitted to the Day Hospital with a personal schedule of ERT (weekly or every other week) or followed-up every 3–6 months because of treatment at home.

In this study, we included 15/33 patients who accepted to undergo our interview. There were 9 females and 6 males with age ranging from 3 to 40 years. Seven of them were younger than 12 years.

Ten (66%) had Pompe disease (PD; 2 early infantile type (EOPD) and 8 late-onset type (LOPD)). The sample also included 2 patients with Mucopolysaccharidosis type IV (MPS IV), 2 pediatric patients with Gaucher disease, and 1 adult subject with Fabry disease. All participants were receiving iv ERT (alglucosidase alfa, elosulfase alfa, imiglucerase, or agalsidase beta, according to their disease). At the beginning of COVID-19 emergency, study patients with Gaucher disease or Fabry disease were on home therapy.

An ad hoc structured interview was developed and administered by phone and when possible by video calls (Table 1) during the third week of lockdown. The interview investigated personal feelings, familial relationship, degree of faith in others, and future perspectives and was inspired and developed in light of this extraordinary, life-threatening event. Quantitative data were obtained from dichotomous questions (Yes/No) used for a clear distinction of respondents’ opinions.

According to the age, we got direct information from 8 subjects, while for 7 pediatric patients, the parents were asked to respond to the interview. A psychologist (GL) from the Centre contacted the patients or their caregivers to assess how the COVID-19 emergency modified the daily life of patients and their family, which changes were due to the resulting Government restrictions, how these were felt, and if any change had occurred with the personal therapy schedule. Moreover, we gathered information about the mood of the patients, their families, and social relationships, the need for psychological support, and their expectations for the future. Since we thought and developed the interview in light of this extraordinary event, the tool could not have been previously validated. To overcome this issue, a group of healthy volunteers was carefully selected for comparison. The control group included 15 healthy subjects matching one-to-one with the patient and caregiver sample, in terms of age, social condition, instruction level, and family composition. 

### 2.2. Statistical Analyses

Data were presented as absolute frequencies and/or percentages for categorical variables. A comparison of proportions between groups was conducted by Chi square test with Yates’ correction. Differences with *p* ≤ 0.05 were considered to be significant. Data were analyzed using the SPSS software, v. 23. (*SPS*, Bologna, Italy)

## 3. Results

### 3.1. Familial Relationships 

Relations with family members appeared to be felt positively in 54% of patients stating that, being at home, they were closer and linked to each other in a co-working and beloved environment. On the contrary, before the lockdown, family members were less involved; moreover, the use of video calls and socials allowed contact with less frequently seen relatives and increased reciprocal affection and the feeling to be part of the same family. In the control group, a positive evaluation was found only in 30%, although they also stated to have rediscovered human values and lost values. 

A negative feeling was reported by 33% of our patients: they described intolerance, impatience, discomfort, distress, constriction, and impairment of contact with close relatives, if not by video calls. In contrast, 60% of the control group described a negative feeling of familial interrelationship because of isolation, uncertainty, fear, difficulties in handling children, and anxiety for older relatives with whom it was hard to communicate. 

A small percentage of investigated patients (13%) and 10% of the control group denied significant changes, stating that they were used to this aloneness and isolation. 

As a whole, no significant differences were observed in the rate of subjects experiencing positive, negative, or unchanged familial relationships between groups (*X*^2^(2, *N* = 25) = 1.7, *p* = 0.413).

### 3.2. Social Relationships 

Patients revealed a strong inclination to feel “others” negatively (87%), as other people were considered to be disrespectful, self-oriented, or dangerous and were to be avoided. Thus, relationships were commonly seen as characterized by lack of empathy, indifference, and detachment. In the control group, we also found a clear tendency to perceive other people negatively (80%). However, in the control group, the image of “others” was that of insecure, frightened, suspicious, avoidant, and elusive people, although considered only slightly inaccessible and deserving of being turned away. 

A small percentage (6%) of our patients, on the contrary, stressed the empathic attitude toward others who were then sharing the common fragility state. In the control group, 20% reported social relations positively stating how useful it was to protect each other by avoiding contact and discovering new ways of social interaction even with neighbors. Only 7% of patients stated that they did not feel significant changes. The proportion of participants who experienced social relationships as dangerous or positive was not significantly different between groups (*X*^2^(1, *N* = 25) = 0.7, *p* = 0.068).

### 3.3. Daily Routine changes 

No significant differences between groups were observed in the rate of participants experiencing negative or positive reactions to modified daily activities (*X*^2^(1, *N* = 25) = 0.3, *p* = 0.802). 

Sixty percent of patients described boring moments, monotony, weakness, and stress for web lessons, limitation of normal activities, prohibitions in moving to familial places and seeing relatives, and the need for repetitive hand hygiene procedures. Likewise, the majority of control subjects (80%) demonstrated a negative reaction regarding the monotony of daily life, which was felt as difficulty in commitment to following rules, in the need for space and temporal organization, and in the occurrence of sleep–awake rhythm problems.

On the contrary, 20% of patients stated that they felt more relaxed and helped by the family dedicating more time to them. In addition, 20% of controls felt the changes positively, having more free time for themselves and for their domestic activities. Among patients, 20% felt that there were no changes in their daily routine.

### 3.4. Personal Feeling and Emotional Reactions

Mostly negative feelings were encountered in our patients’ sample; 67% experienced fear, distress, anger, frustration, impotence, negative mood, and feeling of neglection; and 33% showed ambivalent emotions, with co-existence of astonishment, confusion, doubt, curiosity, and uncertainty alongside the need to protect their beloved ones.

Seventy percent of controls manifested aloneness, anxiety, concern, fear of the unknown, fear of contamination, sharing difficulties, pessimism, mood depression, sadness, and feeling of being in a surreal condition. On the contrary, 20% had a positive mood characterized by adaption, respect, positive dependence, and ability to find incitements and new energies; the remaining 10% showed ambivalent aspects with a hard and pessimistic approach, despite a feeling of well-being. In sum, the proportion of subjects suffering negative or positive feelings was not significantly different in the two groups (*X*^2^(1, *N* = 25) = 0.2, *p* = 0.902).

### 3.5. Relationships with Authorities 

Patients with LSDs expressed their belief in state, regional, and hospital Institutions in 67% of cases; 20% declared to be not confident; and 13% were uncertain because of discordant news and lack to timely assured protection devices. Similar results were obtained in the control group with 70% manifesting faith, 20% manifesting diffidence and the feeling to be abandoned, and 10% showing an uncertainty to judge about the emergency-handling strategies (*X*^2^(1, *N* = 25) = 0.1, *p* = 0.986).

### 3.6. Psychological Defence Mechanisms

Defense mechanisms adopted by patients and controls during the COVID-19 emergency were analyzed: both groups tried to use mature psychological defenses (33% versus 36%, respectively) or denied any concern (11% of patients versus 7% of controls); annihilation was encountered in 11% of patients and 15% of controls; a tendency to discredit others was present in 11% of patients and 14% of controls; some of the patients (17%) activated distressing and pacifying actions; this was also seen in 7% of controls. A small percentage of patients (11%) showed a passive mood, demonstrating lack of affective interactions. 

Almost half of the patients thought that, from this experience, they learnt something positive (47%) such as the real meaning of relationships, gratitude, and the ability to accept and respect others and to identify priorities. This feeling was even stronger in the control group (70%), stating that some positive aspects were coming from the actual situation as the discovery and enforcement of community spirit, sense of belonging to the same community and nation, values of life, solidarity, and the ability to face hard tasks and overcome limits gave a look inside themselves. Twenty percent of patients stated that they were living this experience in a negative way, learning disillusion, frustration, and resignation to death; 10% of controls lived this dramatic situation as subverting daily routine and forcing to reschedule life; 33% of patients stated that there was nothing to learn by this situation, but to just wait for improvement; and 20% of controls were not able to cope with the actual moment. The proportion of subjects who reported to have learnt positively or negatively from this experience did not differ between the groups (*X*^2^(1, *N* = 25) = 1.3, *p* = 0.5118).

### 3.7. Future Perception

Almost half of the patients (53%) manifested negative anticipations, forecasting more preventive precautions, difficulties, limitations, and discomfort than those that had already suffered because of the disease, thus passing to resignation and unavoidable acceptance. Such a negative future perception was present in 50% of the control group, forecasting a sad, stressing, and financially hard future, with consequences on work and relationships. Nevertheless, 34% of patients had a positive vision of the future, including the opportunity to come back to normal life, due to a profound faith in scientific research. Fifty percent of controls prefigured the return to normal daily life, although with unavoidable changes in physical relationship and environment. Among the patients’ group, 13% had a passive, static attitude without changes in future perspectives. No significant differences were observed between groups (*X*^2^(1, *N* = 25) = 1.3, *p* = 0.5118).

### 3.8. Therapy Changes

Most patients (60%) refused to regularly come to the Hospital for their therapies because they feared that they would be infected. The remaining 40% respected their scheduled infusion in the Hospital, although they expressed their fear to be infected and thus showed a strict adhesion to hygiene procedures. All patients asked to be treated at home, except for a child that was severely affected with Pompe disease, whose parents felt safer coming to Day Hospital , but accurately checked the personnel health state. The fear of contamination was also observed in patients who had already been treated with home therapy as they were scared to allow people to come home.

## 4. Discussion

The Italian Government’s emergency declaration on 9 March, 2020, drastically changed our lives. Every action, behavior, or even gesture was filtered by the COVID-19 alert. “Stay at home” was mandatory for all people, except for medical doctors, all health operators, and patients needing urgent medical care (www.salute.gov.it).

This study emerged from the observation of different reactions in patients with lysosomal disorders dealing with pandemic outbreak. LSDs are genetic, multisystem diseases [2]. To date, some of the rare LSDs are susceptible to ERT, which has been shown to at least delay progression, allowing a better quality of life in terms of disabilities. Although, we offered a regular and COVID19-controlled service for these patients, who deserved to be regularly treated despite the emergency period, we observed that patients and their parents were extremely scared and worried about coming to the Hospital, fearing a higher risk for COVID contamination. Thus far, most of them missed their scheduled ERT. Conversely, those patients who had already been treated with home therapy refused treatment by the dedicated team because they felt that this could represent a potential source of the infection.

In this regard, Sechi et al. (2020) analyzed data collected by a questionnaire from 102 patients on ERT therapy for LSDs in Italy [5]. They found that almost 50% of patients who were receiving therapy at a hospital (61.8%) had disruptions, especially for personal feelings (fear of infection).

In the present study, we analyzed behavioral and emotional profiles of our patients with LSDs during the pandemic, compared to healthy controls. For this reason, a structured interview was created and administered online by a trained psychologist, already known to all patients as working in the Centre. Since we thought and developed the interview in light of this extraordinary event, the tool could not have been previously validated. The interview was made during the first week of lockdown, when both patients and controls were experiencing the same uncertainty resulting from the special situation. This study certainly may have some limits due to the small sample size, but this is the rule dealing with patients with rare diseases who are referred to a single center. Although no significant quantitative differences were observed in the type of response between clinical and control groups, there were some qualitative differences between the two groups in all investigated areas. 

In general, all people forced to stay at home and freeze their jobs and who were far from common relationships, but too close to the familial nucleus members, experienced an increase in anxiety, worries, and uncertainty fostered by incessant media updates on virus lethality and virulence, underlining its invisible presence in our environment and in our lives.

Nevertheless, peculiar differences emerged in social relations and perception of “others”. The clinical group, always accustomed to dealing with diversity, exclusion, and unawareness of others with respect to their lives of illness, perceived “others” mostly in a negative way, as dangerous, disrespectful, and not empathetic and to be avoided. The control group, although having the same negative perception, experienced “others” as frightened, insecure, suspicious, and elusive.

In the clinical group, daily routine was marked from the issues of illness and its treatment, while for the control group, main problems were related to difficulty in maintaining, according to an orderly sequence, rules, spaces, times, and sleep–wake rhythm. 

On the other hand, it is singular that, in the percentage of positive representation of the routine changes, subjects in the clinical group believed that the positive element was the opportunity of a better relationship with family members, while among controls, positive elements relied on the possibility of being alone and doing something for themselves. 

Data about future perception evidenced that, while the control group directed its attention to a future focused on economic, social, and environmental issues, patients prefigured a future always oriented by their critical situation being aware of their limits, in terms of treatment opportunities and life expectation. 

We focused on the COVID-19 pandemic effects on medical care and health status of patients with LSDs. The real risk of contagion once again highlighted the vulnerability of patients with chronic rare diseases such as LSDs, the difficulty of coping with his or her defenses, and the need to trust and rely on others. In this case, the subjects preferred to refuse treatment, the only chance to improve their condition. They gathered all their defenses and tried to put in place all the resources and strategies available, such as resist and wait, for example, for home treatment, rather than face unarmed an unknown, enigmatic, and dangerous “enemy”. 

In our sample, a striking similarity emerged between the two groups, equally forced to stay at home and experience the same profound feeling to be excluded (Isolation and Inclusion, *The Lancet Psychiatry* 2020) [6] by normal life. The pandemic, which represented a scary event, suddenly occurring in the daily life, destabilizing, and giving rise to uncertainty, had the same impact as a diagnosis of a chronic rare disease. 

The control group experienced the feeling to be involved in a mutual fight against a common enemy, thus enhancing brotherhood with others. The patients with lysosomal disorders and their families felt that COVID-19 opened the curtains, revealing their human condition of chronic exclusion and impairing their liberty to go out, walk, meet others, love, and breath without fear of death as all the others normally do. Especially the mothers of our patients reacted with strength and determination, feeling that other people can know understand their withdrawn lives to assist their sons. They could now teach others how to face isolation with dignity, aware of fragility, as they usually do: “I can’t help from sadly smiling when my neighbors complain because their children are sad as they cannot go outside…don’t say it to me, please, my child and I do not go out since he was born and he is 4 years old. Now we are all the same, all confined at home… you are not different from us, you also are vulnerable, now we all fear death”. 

The outbreak of COVID-19 evidenced the vulnerability of the patients with such rare diseases and their needs in terms of adhesion to the therapy schedule. Thus far, a special license for home therapy was approved by AIFA (Agenzia Italiana del Farmaco (Italian Medicines Agency), DET. 341/2020), including those drugs prescribed and dispensed only at hospitals. This determination allowed home treatment for most of our patients. The COVID-19 emergency revealed LSD patients’ strength in terms of improved relationships, such as adhesion to the patients’ group, family members, and community and their observance of imposed rules and precepts, trust in authority and doctors, and hope for improvement.

## Figures and Tables

**Table 1:** Structured interview (topic guide).

Name (Initials) Sex Age School degree Profession Marital status Diagnosis Age at onset Age at diagnosis Age at start of ERT Number of members in the family unit How do you live family relationships? How have they changed? Which is your perception of the “Other” How do you spend your time? Any changes because of pandemic? Changes in daily routine Changes in Hospital routine for therapy? What do you think about the current situation? What this pandemic is teaching you? Which is your prevailing emotion at the moment? How do you live the current situation? Confident/not confident in others Confident/not confident about web news or official news Defense mechanisms/coping strategies What is your vision on the future prospects of health and work?
